# A preliminary study on effects of storage time and repeated freezing-thawing on the stability of avian serum amyloid A

**DOI:** 10.1186/s13028-024-00764-8

**Published:** 2024-09-02

**Authors:** Haerin Rhim, Chaeyoung Kwag, Jae-Ik Han

**Affiliations:** 1 Laboratory of Wildlife Medicine, College of Veterinary Medicine, Jeonbuk National University, Iksan, 54596 Republic of Korea; 2 Jeonbuk Wildlife Center, Jeonbuk National University, Iksan, 54596 Republic of Korea

**Keywords:** Acute phase protein, Bird, ELISA, Long-term, SAA, Wildlife

## Abstract

Within the field of clinical research, reports on the stability of avian serum amyloid A (SAA) under varying storage conditions are currently scarce. In this study, avian plasma samples were evaluated for SAA, a major acute-phase protein in birds, to assess how varying storage periods and repeated freeze-thaw cycles impact the stability of SAA in the frozen samples. Seven plasma samples from two species and six plasma samples from three species stored at ‒20 °C were used to evaluate the time and temperature effects accordingly. A chicken-specific SAA ELISA kit was used for the measurements. Statistical analysis was performed using SPSS, and the Kruskal-Wallis test and Spearman’s correlation coefficient were applied, with statistical significance set at *P* < 0.05. The SAA concentrations measured daily for 30 days showed no statistically significant differences over time. Freezing-thawing was repeated five times, and a significant negative relationship was confirmed over the cycles (*r*=‒0.8857, *P* < 0.05). Although no significance was observed between a decreased concentration and the number of cycles, a decrease in the concentration of > 10% was observed after the fourth cycle in four out of six samples. However, one to three freeze-thaw cycles did not result in a significant decline. Taken together, the results indicate that a negative correlation existed between the mean concentration and multiple freeze-thaw cycles, indicating that these should be avoided where possible.

## Findings

The acute phase reaction (APR) serves as a non-specific defensive response to a range of internal or external stimuli, playing a crucial role in maintaining physical homeostasis [1]. In response to adverse immunological events, the expression of acute phase proteins (APP) is significantly elevated through the action of cytokines [2]. While these proteins typically exist in the blood at minimal levels, their expression undergoes substantial changes during the APR, leading to their widespread use in medical and veterinary screenings [3]. Although APP are not in themselves indicative of a specific disease, they are employed, alongside other diagnostic tests, to narrow down potential causes.

Major APP have been identified in both humans and various animals, and their measurement assay is a routine procedure in a clinical setting for humans and animals alike. This testing is extending its application beyond companion animals and livestock to various wild animals [4–8]. In the avian context, proteins like serum amyloid A (SAA), alpha-1-acid glycoprotein (AGP), haptoglobin, and transferrin have been mainly described in chickens, falcons, and geese [9–13]. Among these, SAA stands out as a hydrophobic apolipoprotein of high-density lipoprotein with several isoforms [14, 15]. It exhibits high sensitivity, increasing rapidly during APR, up to 100–1000 times in both humans and animals [16–19]. While the precise maintenance period varies among animals, peaks tend to occur within 24–72 h, followed by a rapid decrease [20, 21]. SAA plays a crucial role in inflammation regulation, although it was initially identified as a fibrillogenic precursor of Amyloid A protein [3, 22].

Despite these insights, reports on the use of SAA as a clinical indicator in wild birds remains lacking. To date, there are still no commercially available reagents or testing kits that can be utilized in various birds, and the only ELISA kit available is for chickens. While testing fresh samples immediately is ideal, real-time testing with a single sample is limited due to the technique’s nature, necessitating storage. Also, stored samples can be tested in various cases, including validation of a method and tracking changes by comparing past samples with the present [23, 24]. This study aimed to fill this gap by evaluating the stability of SAA under varying storage conditions before its widespread application in wild birds. Our hypotheses were: (1) SAA concentrations would not change significantly during 30 days at ‒20 °C; (2) SAA concentration would decrease with repeated freeze-thaw cycles.

Several studies have been based on reports that APP, including human SAA, remain stable when stored at ‒20 °C [25, 26]. However, freezing can damage proteins due to surface denaturation and increase turbidity after thawing which may interfere with analysis [27]. In another study, AGP was found to be not affected by freezing [28]. Similarly, serum amyloid P, known for its role in human amyloidosis, showed no significant change when stored at 4 ℃ or ‒30 ℃ for four weeks, even after three freeze-thaw cycles [29]. In horses, one study found no significant change in SAA values of samples stored in a refrigerator for two months [30]. Another study reported consistent concentrations of equine SAA in both room-temperature and refrigerated samples for up to 17 days after collection [31]. On the contrary, frozen bovine serum samples exhibited a significant decrease in SAA levels starting from the second day of storage, suggesting refrigeration over freezing [32].

Plasma samples were collected from rescued wild birds (*n* = 13) at the Jeonbuk Wildlife Center during intake examinations. A manual complete blood count was performed immediately, followed by biochemical tests after plasma centrifugation. To avoid any changes being masked by a low baseline value, birds with inflammation confirmed by blood test results, including elevated white blood cell count and toxic changes predicting high SAA, were selected. The SAA was measured using an anti-chicken SAA ELISA kit (Eagle Biosciences, Amherst, NH, USA). All samples were diluted 50:1 prior to measurement, according to the manufacturer’s instructions. This kit was validated in our previous study and was used after preliminary tests were performed to evaluate whether the same sample in the three species yielded a consistent measurement across serial dilution ratios [23, 33]. All measurements were performed in duplicate and blinded to sample information. The intra- and inter-assay variations were 3.35% (range 0.22–16.23%) and 8.68% (range 8.3–9.06%), respectively. The lowest detection limit (mean + 2*SD) was 0.072 ng/mL.

To evaluate the effect of storage time on SAA, seven samples from feral pigeons (*Columba livia*) and Eurasian eagle-owls (*Bubo bubo*) were examined. Plasma was divided into microtubes and stored at ‒20 °C, with the first test conducted before freezing. Measurements were performed at the same time daily for 30 days at room temperature. For the effect of freeze-thaw cycles, six samples from common kestrels (*Falco tinnunculus*) and feral pigeons were examined. Plasma was divided into microtubes in aliquots and stored at ‒20 °C, and the first test was performed before freezing. The freeze–thaw cycle was repeated five times. The samples were frozen for at least two days and kept at room temperature for 30 min to be completely thawed before measurement.

Storage stability was evaluated using Spearman’s correlation coefficient and the Kruskal-Wallis test. Post-hoc tests were performed using the Mann-Whitney U test with Bonferroni correction. The Mann-Whitney U test was also applied separately to compare the days. Statistical significance was set at *P* < 0.05, using SPSS V27 (IBM SPSS, Armonk, NY, USA) and GraphPad Prism V9 (GraphPad Software, San Diego, CA, USA).

**Fig. 1:**
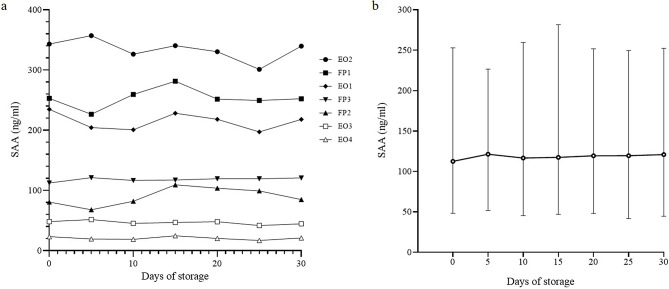
Plots of SAA concentrations for 30 days storage at ‒20 ℃. The graph (**a**) shows each value in the seven samples over time. No significant change according to the days was observed. (**b**) Plot of the median SAA concentration of all seven samples. The median (dots) and interquartile range (bars) are shown. No significant change between the days was observed. EO, Eurasian eagle owl; FP, feral pigeon. SAA: Serum amyloid A

**Fig. 2:**
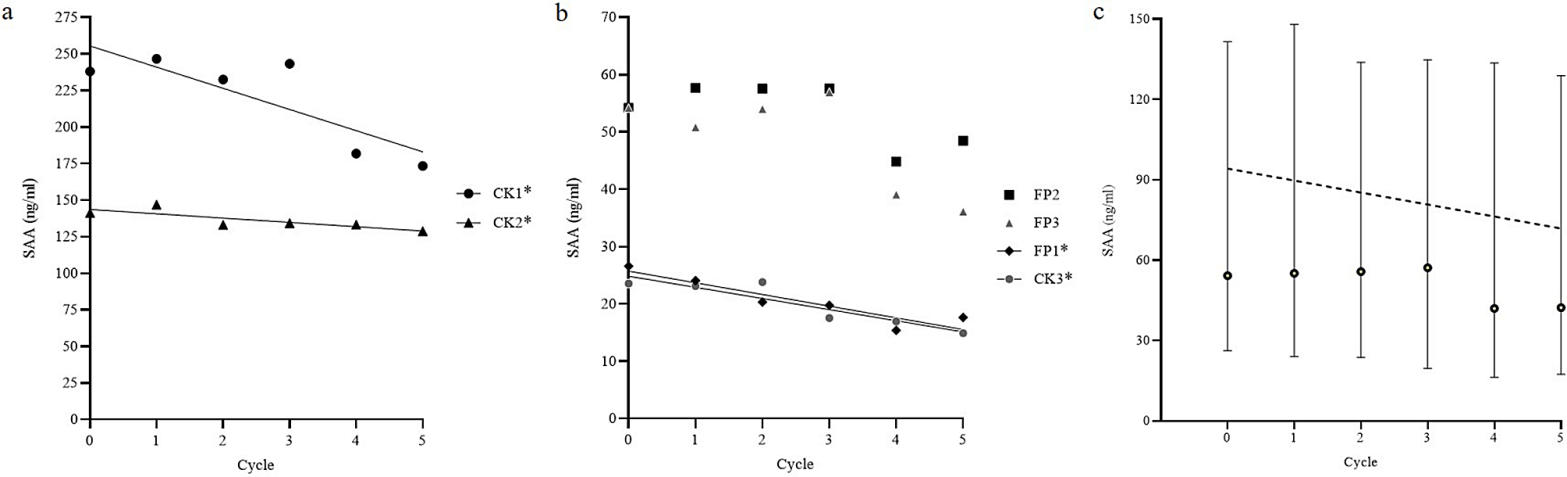
Scatter diagram and linear regression of SAA concentrations following repeated freezing and thawing cycles. (**a**) Two samples with high values; (**b**) four samples with moderate values. (**c**) Plot of the median SAA concentrations of all six samples over cycles. The median (dots), interquartile range (bars), and simple linear regression (dotted line) are shown (*r* = ‒0.8857, *P* < 0.05). Asterisks (*) denote samples showing a significant negative correlation with mean concentration after repeated freeze-thaw cycles. CK: common kestrel; FP: feral pigeon; SAA: serum amyloid A

No statistically significant change was observed in the SAA concentrations of the seven plasma samples during the 30-day storage period (Fig. 1). Their variance was within the CV range of 10%. A significant negative relationship was observed between the mean SAA concentration in response to freezing and thawing, according to Spearman’s correlation coefficient (*r*=‒0.8857, *P* < 0.05) (Fig. 2). The same result was confirmed in four out of six samples when analyzed individually (‒0.935 < *r*<‒0.651, *P* < 0.05). A tendency to decrease with repetitions was found in the freeze-thaw processes from the third to the fifth cycle. The value decreased by over 10% from the second cycle in one sample to the third cycle in one sample and to the fourth cycle in three samples, which was out of the acceptable range for the method used. However, the analysis of which cycle showed a significant decline was not statistically effective for all samples.

Plasma or serum samples are often stored before testing due to limited in-clinic equipment, inability to run a test immediately, or having only one sample. However, the choice of storage temperature and period has been reported to affect the samples and their subsequent analysis results [34]. Changes in many serum biochemical parameters in response to storage conditions have been studied in both humans and animals [35–39]. In general, serum is stored frozen because the levels of many analytes can be significantly altered when refrigerated for an extended period. Frozen samples stored at ‒20 ℃ did not show any significant alterations in SAA values during the 30-day study, unlike one study in bovine SAA [32]. This might be due to the different effects on protein structure depending on the speed of the freezing and thawing process [40]. Whether this is due to differences in animals, requires further investigation as the sample numbers in both studies were small.

A previous study reported marked fractional changes in plasma protein electrophoresis from the second day of refrigeration of psittacine samples, reflecting a much weaker stability than freezing [41]. However, it was also reported that refrigerated samples were also stable for several days, suggesting that a comparative study at different storage temperatures is warranted to determine whether SAA is affected by refrigeration or freezing [30, 31]. Additionally, since samples older than several months or years might be used in practical situations, samples stored for a longer period still need to be warranted.

On the contrary, we could confirm the negative relationship between the freezing-thawing cycle and avian SAA. Although not all samples showed a statistically significant negative correlation in individual analyses, a decrease of > 10% from the initial value was observed in all of them after the fourth cycle. Nevertheless, when the number of cycles was less than four, the values were within the acceptable variation (< 10%). Similarly, no significant change was observed in the fraction percentages of psittacine plasma and canine C-reactive protein stored at − 20 °C according to repeated freezing and thawing up to three and four times, respectively [24, 41]. The fact that decreases after the fourth cycle were observed in five out of six samples were indicative that repeated freeze-thaw should be avoided as far as possible. However, freeze-thaw from one to three cycles did not appear to have a significant effect on the concentration of avian SAA.

As a limitation of our study, we used birds with confirmed inflammatory responses in this study. Even though there was an inflammatory reaction confirmed by blood smears, it was unknown how much the SAA level would have risen. However, given that the CV of ELISA methods is within 10%, low SAA levels could mask detectable changes, even if the daily variation was not predictable. It was confirmed in a later study that SAA values used in this study increased from mild to severe [33], but due to sampling bias, a broader survey including healthy birds should be addressed in the future.

In conclusion, SAA was found to be stable for one month and 1–3 times of freeze-thaw cycles in plasma frozen at ‒20 °C. However, a negative correlation was observed in samples over repeated freeze-thaw cycles. These findings contribute to the understanding of SAA’s reliability in avian health assessments and emphasize appropriate sample handling for accurate results.

## Data Availability

The datasets used and/or analyzed during the current study are available from the corresponding author on reasonable request.

## Abbreviations

AA: Amyloid A
AGP: Alpha-1-acid glycoprotein
APP: Acute phase protein
APR: Acute phase reaction
SAA: Serum amyloid A

## Declarations

### Prior publication

Data have not been published previously.
